# The Welfare Implications of Using Exotic Tortoises as Ecological Replacements

**DOI:** 10.1371/journal.pone.0039395

**Published:** 2012-06-19

**Authors:** Christine J. Griffiths, Nicolas Zuël, Vikash Tatayah, Carl G. Jones, Owen Griffiths, Stephen Harris

**Affiliations:** 1 School of Biological Sciences, University of Bristol, Bristol, England; 2 Mauritian Wildlife Foundation, Vacoas, Mauritius; 3 Institute of Evolutionary Biology and Environmental Studies, University of Zurich, Zürich, Switzerland; 4 Durrell Wildlife Conservation Trust, Les Augrès Manor, Trinity, Jersey, Channel Islands; 5 Bioculture Ltd., Senneville, Rivière des Anguilles, Mauritius; Australian Wildlife Conservancy, Australia

## Abstract

**Background:**

Ecological replacement involves the introduction of non-native species to habitats beyond their historical range, a factor identified as increasing the risk of failure for translocations. Yet the effectiveness and success of ecological replacement rely in part on the ability of translocatees to adapt, survive and potentially reproduce in a novel environment. We discuss the welfare aspects of translocating captive-reared non-native tortoises, *Aldabrachelys gigantea* and *Astrochelys radiata*, to two offshore Mauritian islands, and the costs and success of the projects to date.

**Methodology/Principal Findings:**

Because tortoises are long-lived, late-maturing reptiles, we assessed the progress of the translocation by monitoring the survival, health, growth, and breeding by the founders. Between 2000 and 2011, a total of 26 *A. gigantea* were introduced to Ile aux Aigrettes, and in 2007 twelve sexually immature *A. gigantea* and twelve male *A. radiata* were introduced to Round Island, Mauritius. Annual mortality rates were low, with most animals either maintaining or gaining weight. A minimum of 529 hatchlings were produced on Ile aux Aigrettes in 11 years; there was no potential for breeding on Round Island. Project costs were low. We attribute the success of these introductions to the tortoises’ generalist diet, habitat requirements, and innate behaviour.

**Conclusions/Significance:**

Feasibility analyses for ecological replacement and assisted colonisation projects should consider the candidate species’ welfare during translocation and in its recipient environment. Our study provides a useful model for how this should be done. In addition to serving as ecological replacements for extinct Mauritian tortoises, we found that releasing small numbers of captive-reared *A. gigantea* and *A. radiata* is cost-effective and successful in the short term. The ability to release small numbers of animals is a particularly important attribute for ecological replacement projects since it reduces the potential risk and controversy associated with introducing non-native species.

## Introduction

Conservation introductions such as ecological replacement or assisted colonisation, which are defined as the intentional movement and release of an organism outside its indigenous range [1], are increasingly being explored as potential remedies for dysfunctional ecosystems and to reduce extinction risk [2], [3], [4], [5], [6], [7]. Ecological replacements are effectively alien species introduced to resurrect ecosystem functions once performed by extinct species, whereas assisted colonisation is primarily undertaken to ensure species survival as protection from current or likely future threats is deemed less feasible in its current range than at alternative sites. Much debate about these controversial strategies has focussed on the impact that the deliberate movement of species to novel ecosystems will have on the recipient environment [8], [9] rather than on the welfare of the animals themselves.

Since the deliberate and mediated movement of living organisms from one area with release in another are types of translocation [1], ecological replacement and assisted colonisation could be evaluated using criteria commonly applied to reintroduction projects. Whilst these often define success as achieving a self-sustaining population [10], this is complicated by funding initiatives of one to three years, which are rarely compatible with the timeframes appropriate for long-term monitoring [11]. So pragmatically a broader range of parameters have been used to measure success e.g. post-release survival to maturity, breeding by the second wild-born generation [12], [13], [14], with the criteria selected determined by the time the assessment is made [15].

Long-term monitoring to assess translocation success is particularly problematic for species with long life histories such as late age at maturation and long generation times, and can be complicated by their ecology [14], [16]. For instance, since many ecological replacement projects involve long-lived species [3], [17], [18] but see [19], basing success on whether they establish a self-sustaining population is not practical within a human lifespan, nor necessarily realistic since ecological replacements may require that the translocated population is maintained at a pre-determined level to have a desired ecological impact. We need shorter-term measures of the success of ecological replacements, given that many of the species involved are themselves endangered and the habitats that they are to restore are degraded [2], [17].

Many translocations have had a low rate of success [10], [13], with high mortality rates of released individuals [20] but see [12]. One factor positively associated with translocation success is the release of animals into their core historical range [10], [21]. Given that ecological replacement involves introducing animals beyond their historic range, and possibly into very different environmental and topographical conditions to those of their present-day range *sensu* [17], an unfamiliar environment could affect health, survival and reproduction [20], [22]. Furthermore, particular aspects of translocation, such as disease screening, captivity, transport and release, can be stressful and increase the overall vulnerability of individuals, thereby decreasing the probability that the population will become self-sustaining [20], [22] and ultimately the effectiveness of ecological replacements in restoring functional processes. All this raises ethical questions as to whether animals should be used in ecological replacements [23] and, since translocations can be expensive [13], whether limited financial resources can justifiably be used on such programmes rather than reintroducing native species and protecting nature reserves [24], [25], [26]?

Two species that have been used as ecological replacements are the Aldabra giant (*Aldabrachelys gigantea*) and Madagascar radiated (*Astrochelys radiata*) tortoises, which have been introduced throughout the Indian Ocean [3], [27], [28], [29]. The taxonomy and systematics of the Aldabran giant tortoise are currently under review by the International Commission of Zoological Nomenclature. Whilst we use *Aldabrachelys gigantea*, previously used synonyms are any of the following four generic names (*Testudo*, *Geochelone*, *Aldabrachelys* or *Dipsochelys*) in combination with any of the three species names (*gigantea*, *elephantina* or *dussumieri*). *A. radiata* and *A. gigantea* reach sexual maturity around 16 and 20 years old respectively, and have estimated life spans of over 100 years [30], [31]. *A. radiata* is critically endangered [32], with extinction in the wild forecast in less than 50 years [33], whereas *A. gigantea* is vulnerable, as wild populations occur only on the low lying Aldabra atoll. This has been repeatedly submerged in the past by rising sea levels, resulting in the extinction of previous tortoise species [34]. Conservation introductions to other Indian Ocean islands could prevent the potential extinction of both species in the wild, whilst simultaneously benefiting the recipient communities in terms of resurrecting missing ecosystem functions. *A. gigantea* was introduced to Ile aux Aigrettes in 2000, and both species to Round Island in 2007, two Mauritian offshore islands, as potential ecological replacements for the extinct Mascarene giant tortoises (*Cylindraspis* spp.) [2]. Ongoing monitoring of the recipient ecosystems is being undertaken to ensure that they perform the desired functions and that unexpected impacts are identified quickly. Evidence to date shows that the introduced tortoises are restoring missing seed dispersal and grazing functions on these islands [5] [unpublished data] thereby helping to restore these degraded ecosystems.

However, to be a cost-effective restoration tool, these tortoises must also be able to adapt, survive and possibly reproduce in their novel environments. Here we evaluate whether the translocations to Ile aux Aigrettes and Round Island have been a success so far based on tortoise survival, health (weight) and breeding success of the first generation on Ile aux Aigrettes. Whilst it is important to consider all four population processes (birth, death, immigration and emigration) that influence whether a population is self-sustaining [35], we focus solely on births and deaths as immigration and emigration to and from these small islands is controlled and manipulated by humans. Furthermore, since there are many demands on conservation budgets [36], and it has been argued that finances should be directed to reintroducing native species [24], [25], [26], we report on the cost of each translocation as recommended by Fischer and Lindenmayer [13].

## Methods

Both *A. gigantea* and *A. radiata* are usually diurnal [30], [37] herbivores, whose broad diets occasionally include non-vegetative matter, such as carrion [38], [39]. As ectotherms, they are active and feed during the cooler times of the day, usually from dawn until mid-morning and from mid-afternoon until dusk [39], [40], [41]. During the heat of the day they shelter in the shade of trees or shrubs. Inability to do so results in heat stress and often death [30], [42]. The ability to drink water through their nostrils means that water can be extracted from shallow pools, making *A. gigantea* and *A. radiata* adapted to arid regions [30], [43].

### Translocation Sites

Ile aux Aigrettes is an open (i.e. controlled public access) nature reserve 700 m off the southeast coast of Mauritius, whereas Round Island is a closed (i.e. no public access) nature reserve 22.4 km off the north coast of Mauritius [44]. The habitat and climate of these two islands are compared to that in the native ranges of *A. gigantea* and *A. radiata* in Table 1. Water accumulation is rare due to the porous coralline bedrock of Ile aux Aigrettes and the steep slopes of Round Island. Small water bodies that do develop rapidly dry out due to sun and wind exposure. Consequently, water was provided as captive tortoises used to water supplied *ad libitum* may experience functional drought conditions on release [45].

**Table 1:** Comparison of habitats in the tortoises’ native ranges and on the Mauritian islands.

	Aldabra	Madagascar	Ile aux Aigrettes	Round Island
Native tortoise species	*Aldabrachelys gigantea*	*Astrochelys radiata*	*Cylindraspis inepta* and*C. triseratta*	*Cylindraspis inepta* and *C. triseratta*
Location	46°20′ E, 9°24′ S [42]	45°07′ E, 25°32′ S [68]	57°73′ E, 20°42′ S	57°47′ E, 19°54′ S
Tortoise habitat	Open mixed scrub, grasslandand low canopy trees [69]	Xerophytic spiny forest. Preferopen habitat with low shrubs,grass cover and understoreyvegetation [30]	Coastal dry forest and scrub,with some open areas [44]	Palm savannah: a mosaic of palms and grassland, interspersed by large areas of rock [44]
Terrain, maximumelevation abovesea level	155 km^2^ flat (8 m above sealevel) coral atoll consisting of4 main islands [69]	Generally flat, but has steep limestone cliffs	0.26 km^2^ coralline flat(12 m asl) island	2.15 km^2^ volcanic island rises 280 m asl. Gentle and steep slopes, some in excess of 30°. Deep gullies common
Mean annual rainfall	1056 mm [70]	<400 mm [30]	1578±288 mm (n = 18 years)*	885±172 mm (n = 4 years)*
Tortoise mass^a^	∼20–30 kg [49]	Female: 5.5 kg(range = 3.1–10.2 kg);male: 6.7 kg(range = 4.5–10.5 kg) [30]	Female: 67–117.5 kg (n = 7);male: 105–192.5 kg (n = 10);subadult: 22.8–37 kg (n = 5);juvenile: 22.8–37 kg(n = 2; Table S1)	*A. gigantea*: 25–40 kg (n = 12);*A. radiata*: 7–14 kg (n = 12)
Potential predators of hatchlings	Coconut and land crabs,white-throated rails, cats,sacred ibis, rats [63]	African bush pigs, Indian civets, snakes, dogs. Also trampled by domestic herbivores (goats,cattle, sheep) [30]	None known	None known
Other vertebrate herbivores in habitat	Goats	Goats, cattle, sheep	None	None

The habitat and climate of the native ranges of *A. gigantea* and *A. radiata* in Aldabra and Madagascar respectively are compared with the offshore Mauritian islands of Ile aux Aigrettes and Round Island, where they were introduced to replace the extinct *Cylindraspis* species.

* Data courtesy of Mauritius Meterological Services.

a Mass when first introduced to Ile aux Aigrettes and Round Island.

Tortoises were penned upon arrival on both Ile aux Aigrettes and Round Island to monitor their health and to allow them to acclimatize before release. A soft release [10] was undertaken to (i) reduce the stress associated with a hard release [20], and (ii) to limit the risk of introducing and spreading exotic seeds that could have been carried enterically.

### Ile Aux Aigrettes

**Figure 1:**
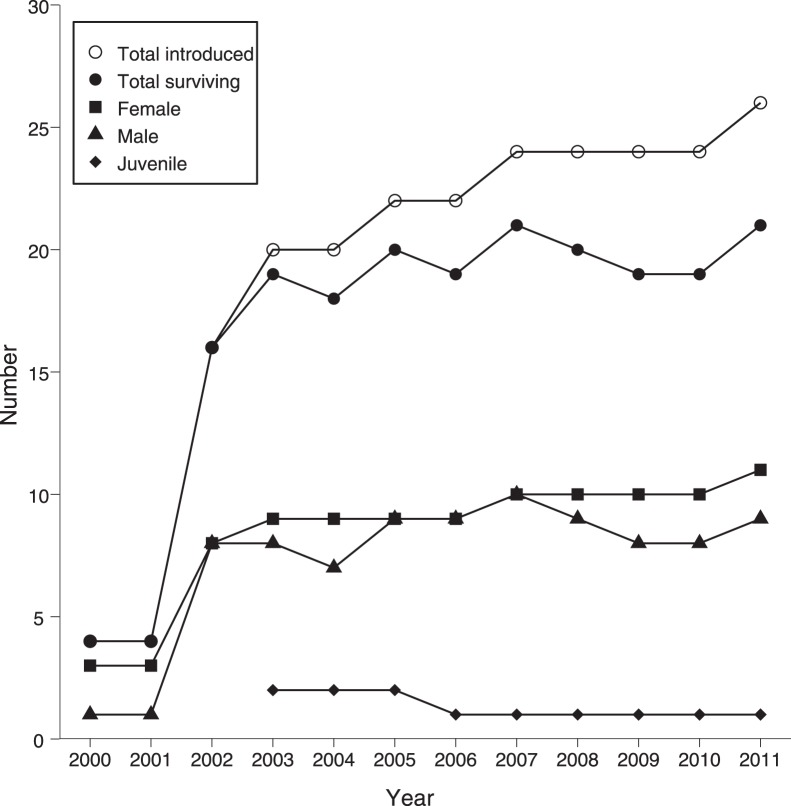
Total number of *Aldabrachelys gigantea* introduced in 2000 and surviving on Ile aux Aigrettes in 2011. Table S1 shows which animals were juvenile, subadult or adult when first introduced. While male and female adult and subadult tortoises are presented separately, the subadults were not sexually mature. Juveniles were those animals which were not sexually mature and whose sex could not be determined.

*A. gigantea* were either loaned or donated by private estates or individuals in Mauritius (see Table S1): many exhibited behavioural problems and were in poor-health upon arrival because they had been reared individually and/or in cramped conditions. *A. gigantea* were kept in quarantine pens for several months prior to and post translocation to Ile aux Aigrettes by motorboat, and treated with fenbendazole to kill intestinal parasites (see Text S1). It is recommended that species introduced for conservation purposes are translocated with their associated diseases [46]. However, these tortoises had been held long-term, often with limited knowledge of their captive histories, and there was a risk that they may have accumulated alien parasites. Since we did not want to risk introducing these to a nature reserve where future reintroductions of endemic reptiles were planned, we took a precautionary approach and treated the tortoises to kill any intestinal parasites. The first four captive *A. gigantea* were moved from their quarantine pen to a 0.01 km^2^ enclosure in November 2000. Additional tortoises were added subsequently (Figure 1), and by late 2004 they were allowed to roam freely. Those in poor health were supplementary fed with either vegetables or vegetation (Table S1).

### Round Island

We conducted a trial introduction of twelve captive-reared juvenile *A. gigantea* (∼8–10 years old; mean weight ± SD = 33±5 kg; mean curved carapace length ± SD = 73.6±9.8 cm) and twelve adult male *A. radiata* (20–30 years old; mean weight ± SD = 10±2 kg; mean curved carapace length ± SD = 55.8±4.0 cm) to Round Island to assess their suitability as ecological replacements. They originated from breeding populations belonging to the Mauritian Wildlife Foundation and La Vanille Réserve des Mascareignes. Juvenile *A. gigantea* were used for logistic reasons and to enable sufficient time to identify negative impacts on the recipient community and to be sure we could remove the whole population before they started breeding, had this proved necessary.

In accordance with International Union for Conservation of Nature recommendations [47], [48], all animals were quarantined and underwent health screening (including faecal, haematological and serological analysis) prior to translocation (see Text S1). While the risk of inter-species disease transmission was low [2], screening was done to ensure that the translocatees were disease-free as Round Island is an important refuge for endemic reptiles [44]. Because *A. gigantea* can have a gut passage time of ∼50 days [49], tortoises were quarantined in concrete pens and fed a restricted diet from 12 March to 26 June 2007 to reduce the risk of introducing exotic plant seeds that may have been retained internally.

Once certified as healthy, the tortoises were translocated by helicopter to Round Island on 27 June 2007. Conspecifics were introduced in pairs into 8×8 m enclosures to monitor each species’ impact on the vegetation [2]. They were released 11–12 months later (14 May and 1 July 2008), having first been fitted with a radio transmitter and located and checked between 14 to 26 days per month for the first 7 to 8 months following release and thereafter every two weeks.

### Monitoring

All tortoises were individually marked and/or implanted with passive integrated transponder tags (AVID Inc., Norco, California) for permanent identification. Whilst weight is a reliable indicator of health in tortoises [50], weights were not used as a regular measure of health on Ile aux Aigrettes as growth rate declines with size/age [37], [51] and most of these animals were large adults (Table S1). Tortoises on Round Island were weighed monthly from March 2007 (3 months prior to the translocation) to January 2009, and again from January 2011 onwards. They were not weighed after periods of heavy rain or having been provided with drinking water to avoid weights being inflated by water retention [45]. We used paired t-tests to test for significant weight changes between March 2007 and December 2011. All analyses were performed with R2.12.2 software [52].

Breeding success was only evaluated for tortoises on Ile aux Aigrettes because they were sexually mature and of mixed sex. Whilst confined to enclosures (2000–2004), pits >0.6 m deep were dug to provide females with suitable nesting sites. Any eggs were removed to prevent them being destroyed by other females digging. Eggs were re-buried in soil, either in indoor containers or in an outdoor hatchery. The collection of eggs ceased post-tortoise release: the first hatchlings (<1 kg) *sensu* [40] were discovered at the start of 2005, indicating that animals could reproduce successfully in the wild. All hatchlings found were transferred to the mainland for security and release on Round Island when large enough (>20 cm plastron length). Hatchling deaths in captivity were recorded. We report on the survival of all animals and summarize any behavioural observations which were relevant to their survival and to the success of the translocation.

### Economics of Translocations

We estimated the expenditure of each translocation project i.e. salary, administration, management, and logistical costs, from the start until the end of 2010 and converted all costs to 2012 US$. Existing infrastructure in place on the islands (e.g., accommodation) was not included in the costs.

### Ethics Statement

This study was carried out in strict accordance with the recommendations in the Guide for the Care and Use of Laboratory Animals of the National Institutes of Health and approved by the University of Bristol Ethics Committee (UB/07/005). All animals were under veterinary supervision: minor problems were dealt with *in situ*. Where necessary, tortoises were returned to mainland Mauritius for treatment.

## Results

### Ile Aux Aigrettes Translocation

Of the 26 *A. gigantea* released on the island, 21 remained at the start of 2012 (Figure 1). Two adult males were euthanized because of kidney failure associated with old age and one male was removed due to a prolapsed penis. A female tortoise died three months after arrival from a fatty liver attributed to an inappropriate daily diet of bread and milk prior to her joining the scheme, and a juvenile tortoise disappeared, believed stolen (Table S1).

**Figure 2:**
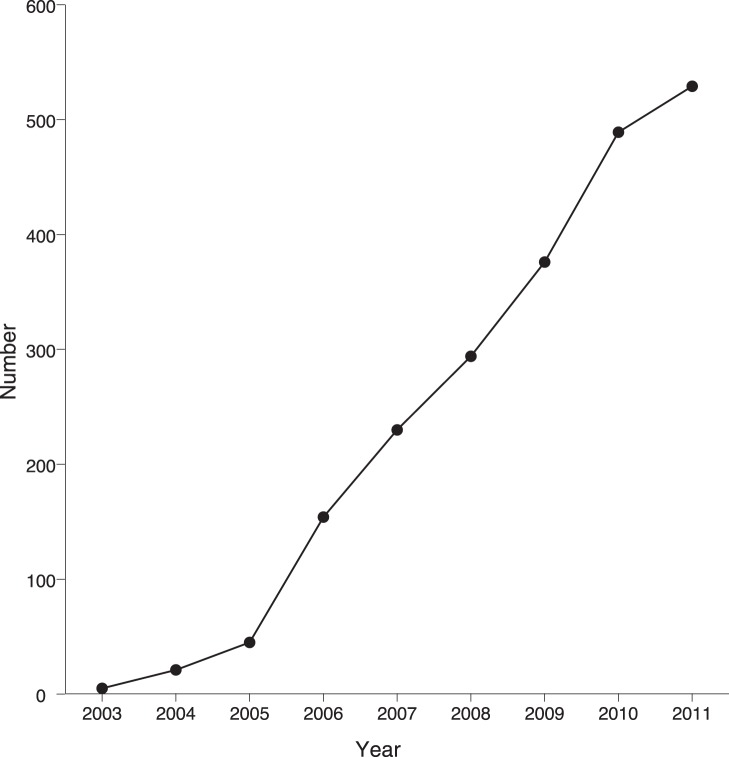
Cumulative number of *Aldabrachelys gigantea* hatchlings obtained on Ile aux Aigrettes between 2003 and 2011.

The first eggs were laid at the end of 2002. Over nine years (2003–2011), we obtained 529 hatchlings (Figure 2); 333 were hatched in the wild, and the rest were from collected eggs. While 99% of hatchlings found were under 6 months old, five were up to two years old. When in captivity on the mainland, 28 hatchlings died, nine from unknown causes, five from heat stress, and 14 from herpesvirus. All sexually mature females were observed laying eggs, whereas early breeding attempts were most commonly observed by the two largest males until after 2006, when new males were introduced (Table S1).

### Round Island Translocation

Of the original twelve animals of each species introduced in 2007, 11 *A. gigantea* and 10 *A. radiata* remained by January 2012. One *A. radiata* was injured during the translocation when the sling carrying the crates with twelve tortoises under the helicopter was released prematurely. The tortoise was returned to the mainland for veterinary attention, where it recovered. No other animal was injured in that incident. An *A. gigantea*, which had not been in the dropped consignment, was returned within 1 month (16 August 2007) due to ill health; it was not eating, moving or defecating. It was unclear why, but after a course of antibiotics the animal regained health. Only one animal died over the four years: one month after release an *A. radiata* was fatally injured after rolling ∼71 m (over 28 m in altitude) down a rock slab. The animal was returned to the mainland for veterinary attention, but died two and a half months later despite appearing to recover.

Overall, the tortoises appeared in good physical health, and the minor problems we recorded were probably typical for free-living tortoises. One *A. gigantea* suffered from an oedema in the neck: this disappeared without treatment. *A. radiata* often had scratches on the thin skin of their neck and around their front shoulders. Grass seeds were also occasionally removed from around their eyes and one *A. radiata* was treated with antibiotics for suspected septicaemia, which may have developed from a cut to its leg.

**Figure 3:**
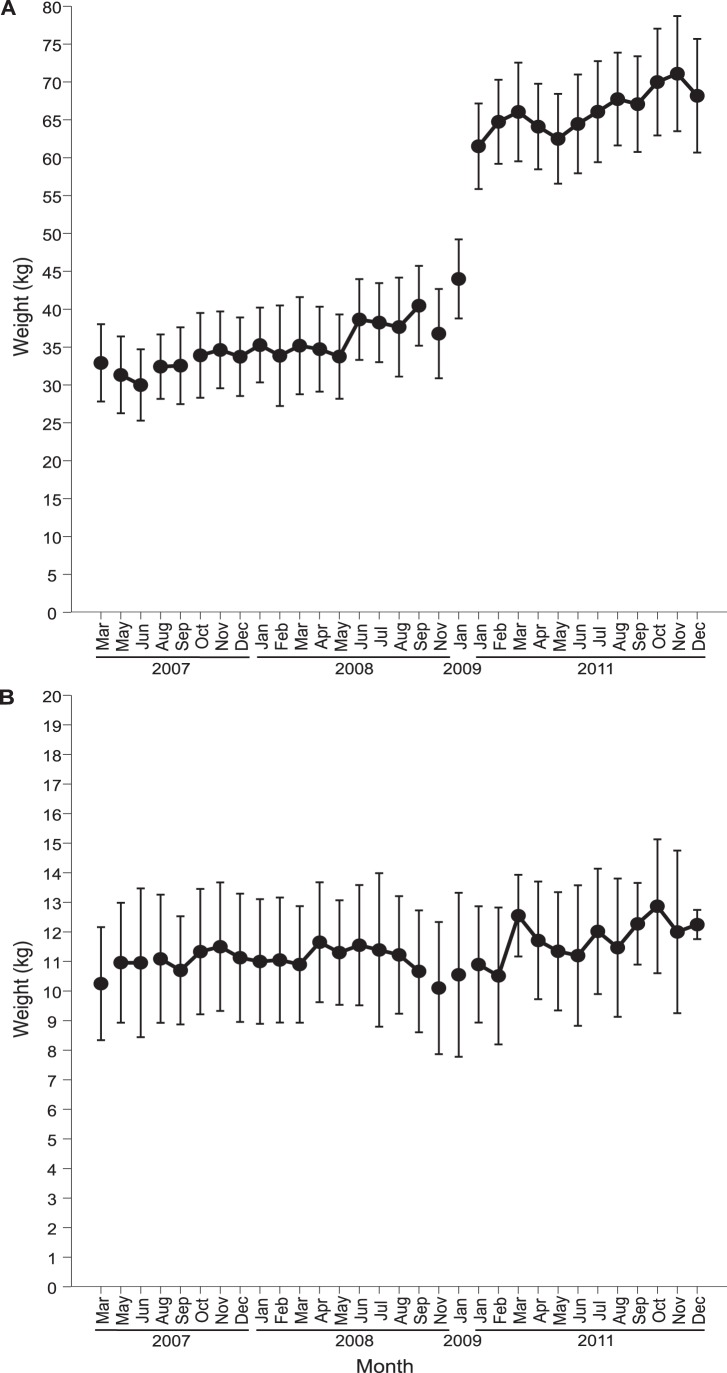
Mean weight and SD of tortoises on Round Island. (A) juvenile *Aldabrachelys gigantea* and (B) adult *Astrochelys radiata* between March 2007 and December 2011.

All the tortoises were significantly heavier in December 2011 than March 2007. Juvenile *A. gigantea* gained 107% of their initial weight (paired t-test, t = −16.57, d.f. = 10, p<0.0001, Figure 3A), while adult *A. radiata* gained 20% (t = −5.11, d.f. = 9, p = 0.0006, Figure 3B). An *A. radiata*, which escaped from its enclosure on the 23 September 2007 and was not relocated until 1 November 2008, maintained a stable weight over those 13 months.

### Other Welfare Indicators

None of the tortoises appeared to be stressed when in quarantine or confined to enclosures. Pronounced urination and defecation during transportation and when taking blood samples indicated that these procedures were stressful. However, once they had reached the islands, adjustment appeared rapid as most animals immediately started feeding. On two occasions, *A. gigantea* on Round Island suffered from heat stress whilst out in the open under the midday sun. They were moved to shade where they recovered.

Although water was provided, tortoises were able to find water on leaf litter or from depressions in the ground. Male *A. radiata* frequently overturned one another while fighting but could right themselves. Site fidelity was common among the *A. gigantea* on Ile aux Aigrettes, with most tortoises initially being reluctant to leave the enclosure when released.

### Economics of Translocations

The introduction and maintenance of a tortoise population on Ile aux Aigrettes cost US$ 38,912 over 11 years, and US$ 32,452 for less than 4 years on Round Island. Average annual costs were lower for Ile aux Aigrettes (US$ 3,537) because all animals were donated or loaned, its proximity to mainland Mauritius meant transport costs were lower, and because disease and health screening was less extensive than on Round Island, where the average annual cost was US$ 8,113 (Table 2).

**Table 2:** Tortoise translocation costs (US $) to Ile aux Aigrettes and Round Island.

	Ile aux Aigrettes	Round Island
Staff (salaries, overheads)	$31,726	$16,057
Transport (staff, tortoises, food procurement)	$3,550	$9,772
Equipment (enclosures, crates, consumables, water catchment)	$3,019	$1,258
Veterinary care (disease screening, consultancy)	$617	$3,678
Tortoises		$1,687
Total	$38,912	$32,452
Number of years	11 (2000–2010)	4 (2007–2010)
Average annual cost	$3,537	$8,113

Costs were converted from Mauritian rupees to US $ using the exchange rate on 27 February 2012.

Staff costs included: the salary of staff to maintain the tortoises in captivity; monitor the tortoises on the islands; employ a researcher on Round Island for 21 months; labour, administration and overhead (e.g. gas, consumables, maintenance of field station) costs.

Transport costs for Ile aux Aigrettes included jeep and boat costs for transporting staff and tortoises; and for Round Island included jeep, boat and helicopter costs for transporting staff, tortoises, and for obtaining food for tortoises when in captivity.

Veterinary care costs involved veterinary consultancy, disease and health screening procedures and removing injured tortoises.

As all tortoises were donated or loaned to Ile aux Aigrettes, no cost was incurred. The cost of tortoises for Round Island only included three animals of each species as nine *A. gigantea* and nine *A. radiata* were donated by La Vanille Réserve des Mascareignes.

## Discussion

Tortoise populations have been managed frequently through translocations, with variable success [28], [45], [53], [54], [55], [56], [57]. Based on our criteria for success, i.e. survival, breeding success of the founder population on Ile aux Aigrettes, and health (weight), the introductions of tortoises to Ile aux Aigrettes and Round Island have been successful to date. We attribute this to the tortoises’ generalist diet and habitat requirements, suitable habitat and innate behaviour [2], [3].

### Mortality and Stress

Mortality rates following an initial translocation are often high. Most reported causes are predators and/or stress, reviewed in [20]. Neither Ile aux Aigrettes or Round Island harboured native or exotic predators of juvenile tortoises, nor were young tortoises at risk of being trampled by other herbivores (Table 1).

While tortoises experienced stress during translocation and disease screening, this was short term *sensu* [22]. The potential for chronic stress, which can be detrimental to the long-term well-being and survival of individuals, was minimised as the animals were: (i) captive-reared and were therefore used to confinement and human contact; (ii) were allowed a period of acclimatization before release; (iii) continued to receive water once released; and (iv) those *A. gigantea* on Ile aux Aigrettes that were in poor health continued to receive supplementary food after release. A hard release, directly into the environment, may have elevated stress levels [20], [58].

Annual mortality rates for *A. gigantea* on Aldabra vary from 1.9–2.1% for tortoises with a curved carapace length over 60 cm [51]. The commonest causes of adult mortality were heat stress and animals becoming trapped or failing to right themselves after having been overturned [59]. In contrast, the average annual mortality rates were 1.3% and 0% for Ile aux Aigrettes and Round Island respectively. It is likely that the mortality rate on Ile aux Aigrettes would have been lower if a younger population had been introduced.

Natural adult mortality of *A. radiata* in Madagascar is very low [30], although anthropogenic mortality, e.g. road deaths, trampling by cattle/goats [41] and illegal harvesting is leading to a rapid population decline [60]. As the terrain where *A. radiata* is found in Madagascar is generally flat, fatal falls are rare. However, tortoises elsewhere die from falling down crevices or sink holes [34], [61] [34], [61], so the fatal fall of one *A. radiata* on Round Island was not unusual.

### Growth Rates and Breeding

Annual growth rates for *A. gigantea* with similar-sized third dorsal scutes (mean ± SD: 23.6±1.3 cm) depend on population density, varying from ∼2.4% on Aldabra to ∼37.4% on the less populated Curieuse Island, where *A. gigantea* were introduced in the 1970s [51], [62]. On Round Island, *A. gigantea* had an annual growth rate of 21.4%, suggesting that the island is a suitable habitat in terms of resource availability [63]. In the absence of other large herbivores, there was ample food available. Indeed, the rationale to introduce tortoises to Round Island was to control the exotic grasses which had flourished following the eradication of invasive mammalian herbivores [2]. As tortoise density increases, growth rates are likely to slow and maximum size decline [51].

The production of hatchlings on Ile aux Aigrettes indicates that the conditions are suitable for *A. gigantea* to breed. Furthermore, the discovery of two-year old tortoises shows that survival extends beyond the first year when mortality is at its highest [63]. While the tortoises are a major tourist attraction on Ile aux Aigrettes, this appears not to have had a visible impact on the animals’ welfare. Tourists are only allowed to visit with an appointed tour guide and must not leave paths. This ensures that egg-laying females are not disturbed, and hatchlings and eggs are less likely to be trampled or stolen. This is important because uncontrolled access by visitors can have a significant impact on *A. gigantea* populations [40] since eggs and hatchlings are vulnerable to being trampled [64]. For example, 250 *A. gigantea*, of which roughly half were female, were introduced to Curieuse Island, Seychelles, in the 1970s and 1980s. Despite producing an estimated 2100–3900 hatchlings between 1978 and 1982, only 34 young were found 12 years after the initial introduction. Poaching for food and the illegal pet trade, the detrimental impact of tourists, and the presence of exotic mammalian predators all contributed to the poor recruitment [28], [40]. In comparison, the twelve female *A. gigantea* introduced to Ile aux Aigrettes produced at least 529 hatchlings in nine years. Although hatchlings are removed, their survival rate is likely to be high *in situ* as there are no predators on the island. Nor is there any evidence that the island’s omnivorous scavengers, the Telfair’s skink (*Leiolopisma telfairii*), crabs (*Cardisoma carnifex*), and the Indian musk shrew (*Suncus murinus*), have predated hatchlings. While it is suspected that hatchlings have been stolen, the presence of a permanent warden and restricted access limit such losses. Overcrowded conditions at the mainland captive holding centre are believed to have activated latent herpesvirus, resulting in hatchling mortality [65]. Tortoises can be carriers of herpesvirus, showing no signs of illness for long periods of time. The source of infection is unknown.

While *A. gigantea* are still too young to breed on Round Island, and all *A. radiata* are male, suitable egg-laying sites exist: soils are sufficiently deep and well-drained, and there is an abundance of open areas with relatively little canopy cover [28], [30], [64]. Whilst hatchlings could be predated by the endemic carnivorous snake (*Casarea dussumieri*), and possibly large Telfair’s skinks, this would also have been the case for the native Mauritian tortoises.

### Versatility of *A. gigantea* and *A. radiata* as Ecological Replacements

New environments are full of survival challenges for any released animal, particularly those that are captive-reared [13]. The problems most commonly associated with releasing formerly captive animals, such as domestication, loss of wild behaviour, loss of resistance to disease, genetic drift, and high financial cost [66], did not apply here. Even though some of the adult *A. gigantea* released on Ile aux Aigrettes had been in captivity for decades, they were still able to adapt to their new environment, and the tortoises released on Round Island adapted to a substantially steeper terrain than they experienced in their captive conditions or occurred in their native ranges (Table 1). The released tortoises were also able to find shade, water, suitable plant species to consume and, on Ile aux Aigrettes, breed for the first time. This is because much tortoise behaviour is innate, their social structure is basic, parental care is nonexistent and they are non-territorial [67]. Furthermore, their generalist diet and habitat requirements probably explain why the transition to a natural diet and to novel environments was successful. In addition, they have the ability to withstand severe injuries [37], [59], long periods with little or no food and water [30], [49], [59], and high degrees of habitat degradation in their native ranges [30]. Because of their hardiness and versatility, it was not necessary to undergo a training period prior to release and using young individuals was not essential to ensure translocation success. Furthermore, for the majority of chelonian species, stress induced by quarantine, transport, and release procedures appears minimal in comparison with that experienced by mammals [22].

While successful translocation is often associated with the release of a large number of animals (n>100) [13], our study shows that releasing small numbers of tortoises as ecological replacements can be successful in the short term, provided the agent for the extinction of the native species has been removed. It also reduces the potential risk and controversy associated with introducing a non-native species.

### Economics of the Translocations

It is essential that ecological replacement projects are cost effective, and the outcomes documented. Our study shows that comparable to other conservation translocations [13], ecological replacements can be low cost. The project costs were low because animals were donated and loaned, technical expertise and facilities required for captive breeding were basic, and minimal long-term intervention was needed. Being social gregarious animals, *A. gigantea* can be housed at high densities, further keeping costs low.In 2010 and 2011, the Mauritian Wildlife Foundation translocated a further 220 juvenile *A. gigantea* (weight range 2–30 kg) to Round Island. They comprised a mixture of the offspring of the Ile aux Aigrettes tortoises and new stock from La Vanille Réserve des Mascareignes to increase genetic variability, as breeding on Ile aux Aigrettes was initially dominated by two males. Releasing animals when younger (3−6 years) will reduce the costs associated with maintaining the animals in captivity for longer periods.

### Conclusions

Although the translocated tortoises have fared well since their arrival, long-term monitoring will be necessary to determine the survival and productivity of these animals without human intervention, and their impact on the recipient environments. *A. gigantea* is vulnerable and *A. radiata* critically endangered and, while this study provides important information about their potential as effective ecological replacements, it also indicates that translocating either species for assisted colonisation or to reintroduce them to native habitats is a viable conservation strategy. Our study provides a useful model of how to conduct and assess other tortoise translocations. In particular, feasibility analyses for ecological replacement and assisted colonisation projects should consider the candidate species’ welfare during translocation and in its recipient environment.

## Supporting Information

Table S1 Weights of *Aldabrachelys gigantea* introduced to Ile aux Aigrettes.
(DOC)

Text S1 Disease screening and quarantining of tortoises prior to being translocated to Round Island.
(DOC)
