# Supplementary material for: The Welfare Implications of Using Exotic Tortoises as Ecological Replacements
Source: PLoS One. 2012 Jun 19;7(6):e39395. doi: 10.1371/journal.pone.0039395 (PMC3378584; doi:10.1371/journal.pone.0039395)
Supplement: Table S1 — Weights of Aldabrachelys gigantea introduced to Ile aux Aigrettes. (DOC) [file pone.0039395.s001.doc]

### Table S1. Weights of *Aldabrachelys gigantea* introduced to Ile aux Aigrettes.

|  |  |  |  |  | **Weight (kg)** | | | | |  |
| --- | --- | --- | --- | --- | --- | --- | --- | --- | --- | --- |
| **Tortoise ID** | **Source** | **Date introduced** | **Sex** | **Age upon arrival**^1^ | **Apr-02** | **Aug-03** | **Apr-04** | **Nov-04** | **Sept-07** | **Comment** |
| Red | Donated | Nov-00 | F | 17 years (SA) |  | 88.5 | 93.5 | 96.5 | 101.5 |  |
| Green | Donated | Nov-00 | F | ~14 years (SA) |  | 72.0 | 70.0 | 71.5 | 77.5 |  |
| Blue | Donated | Nov-00 | F | 14 years (SA) |  | 87.0 | 90.0 | 85.5 | 99.0 |  |
| White | Donated | Nov-00 | M | 14 years (SA) |  | 87.5 | 88.5 | 99.5 | 113.0 |  |
| MT1 | Loaned | Apr-02 | F | >50 years (A) | 67.0 | 72.0 | 76.0 | 79.5 | 77.5 |  |
| MT2* | Loaned | Apr-02 | M | >50 years (A) | 192.5 | 194.0 |  |  |  | Euthanized 2004^2^ |
| MT3 | Loaned | Apr-02 | F | >50 years(A) | 111.5 | 121.0 | 120.5 | 119.5 |  |  |
| MT4* ^a^ | Loaned | Apr-02 | M | >50 years (A) | 165.0 | 140.0 | 152.5 | 142.0 | 125.0 | Euthanized 2008 |
| MT5* ^a^ | Loaned | Apr-02 | M | >50 years (A) | 133.5 | 126.0 | 129.0 | 126.0 | 141.5 |  |
| MT6 | Loaned | Apr-02 | M | >50 years (A) | 186.0 | 196.5 | 188.0 | 195.5 | 206.0 |  |
| MT7* | Loaned | Apr-02 | M | >50 years (A) | 176.5 | 166.5 | 175.0 | 187.0 | 175.0 |  |
| MT8* | Loaned | Apr-02 | M | >50 years (A) | 147.5 | 174.0 | 145.5 | 134.0 | 158.5 | Removed in 2009^3^ |
| MT9 | Loaned | Apr-02 | F | >50 years (A) | 97.0 | 92.0 | 107.5 | 93.0 | 96.5 |  |
| MT10 | Loaned | Apr-02 | M | >50 years (A) | 174.5 | 185.0 | 178.5 | 181.5 | 191.5 |  |
| MT11 | Loaned | Apr-02 | F | >50 years (A) | 70.5 | 78.0 | 87.5 | 83.0 | 86.5 |  |
| MT12 | Loaned | Apr-02 | F | >50 years (A) | 96.0 | 106.5 | 111.5 | 104.5 | 117.5 |  |
| Yellow | Donated | Jun-03 | F | 18 years (SA) |  | 70.5 |  |  |  | Died Sept 2003 |
| L1 | Loaned | Jun-03 |  | 7 years (J) |  | 37.0 | 40.5 | 35.5 | 55.0 |  |
| L2 | Loaned | Jun-03 |  | 5 years (J) |  | 22.8 | 22.8 | 26.0 |  | Believed stolen 2006 |
| Red-green | Donated | Oct-03 | F | 20 years (A) |  |  | 117.5 | 114.0 |  |  |
| R1 | Donated | Sep-05 | M | >40 years (A) |  |  |  |  | 165.5 |  |
| R2 | Donated | Sep-05 | M | >40 years (A) |  |  |  |  | 142.0 |  |
| R3 | Donated | Aug-07 | F | >40 years (A) |  |  |  |  | 78.0 |  |
| R4 | Donated | Aug-07 | M | >40 years (A) |  |  |  |  | 105.0 |  |
| R5 | Donated | Nov-11 | F | >40 years (A) |  |  |  |  |  |  |
| R6 | Donated | Nov-11 | M | >40 years (A) |  |  |  |  |  |  |

Tortoises were classified as adults (A) if they were sexually mature, subadults (SA) if their sex could be determined from secondary sexual characteristics but were not sexually mature, and juvenile (J) if they were not sexually mature and their sex could not be determined.

^*^ Males not sexually active due to ill-health.

^a^ Animals were supplementary fed because they were underweight. Both suffered poor eyesight and were thought to be blind.

^1^ Age of tortoises over 20 years was conservatively estimated.

^2^ Animal was ill when it arrived.

^3^ Animal had a prolapsed penis which was being damaged on the rough coral substrate.
