# Supplementary material for: The Welfare Implications of Using Exotic Tortoises as Ecological Replacements
Source: PLoS One. 2012 Jun 19;7(6):e39395. doi: 10.1371/journal.pone.0039395 (PMC3378584; doi:10.1371/journal.pone.0039395)
Supplement: Text S1 — Disease screening and quarantining of tortoises prior to being translocated to Round Island. (DOC) [file pone.0039395.s002.doc]

### Text S1. Disease screening and quarantining of tortoises prior to being translocated to Round Island.

Tortoises were kept in quarantine for over 3 months (12 March - 26 June 2007) at the Gerald Durrell Endemic Wildlife Sanctuary (GDEWS) in Rivière Noire and at La Vanille Réserve des Mascareignes in Rivière des Anguilles; each species was housed in separate pens with coral substrate and concrete flooring at GDEWS and La Vanille respectively, to ensure that only food provided was available to the tortoises. The pens were covered in shade netting to prevent leaves and seeds from entering. Tortoises were fed on a diet of de-seeded vegetables and foliage.

On 14 March 2007, blood samples (~1 ml) were collected from the brachial vein of each tortoise using disposable 24-gauge 1 inch needles and 2 ml syringes. Two or three blood smears were made per animal, air dried and fixed with 100% methanol. The remainder of the blood was transferred to microhematocrit tubes and centrifuged at 10 000 rpm in a minihaematocrit centrifuge for 3 minutes to separate the blood cells from the total solid. Packed cell volume (the percentage of the red blood cells to the total fluid) and total solid (g/100 ml) were measured using a haemocytometer and a calibrated refractometer. Three measurements were taken for each animal.

Faecal samples were collected from each tortoise and preserved in 70% ethanol. Tortoises were physically examined for signs of ectoparasites and ill-health. The tortoises were also tested for herpesviruses and *Mycoplasma agassizii*, which are often associated with respiratory diseases and are potentially fatal [1]. Two methods were used: (i) the upper palate of each tortoise was wiped with a sterile swab, and (ii) a saline solution was flushed into a naris and withdrawn using a syringe. The withdrawn sample was then sprayed onto sterile filter paper and allowed to dry. All faecal, nasal and blood samples were sent to the United Kingdom with the appropriate Convention on International Trade in Endangered Species of Wild Fauna and Flora (CITES) permits, where applicable, and analysed by M. Waters at the Royal Veterinary College, London. All blood and nasal samples were negative. As roundworms were identified in seven faecal samples, all animals were treated with 5% fenbendazole at a dosage of 50mg/kg prior to departure to Round Island, as recommended by A. Greenwood (International Zoo Veterinary Group).

## References

1. Pasmans F, Blahak S, Martel A, Pantchev N (2008) Introducing reptiles into a captive collection: the role of the veterinarian. Vet J 175: 53-68.
